# Risk Factors And Prognostic Impact of Sleep Alterations in Icu Patients - a Prospective Observational Polysomnographic Study

**DOI:** 10.1186/2197-425X-3-S1-A29

**Published:** 2015-10-01

**Authors:** A Demoule, S Carreira, S Lavault, O Pallanca, J Mayaux, J Delemazure, I Arnulf, T Similowski

**Affiliations:** Groupe Hospitalier Pitié-Salpêtrière, Paris, France; UMRS 1158, INSERM and Pierre and Marie Curie University, Paris, France; INSERM and Pierre and Marie Curie University, Paris, France

## Introduction

Poor sleep is common in intensive care unit (ICU) patients. However, the risk factors and the consequences of sleep abnormalities on the outcome are not well established. This all the more true that many studies have not been based on polysomnography and have been conducted in patients receiving sedation.

## Objectives

The aims of our study were: 1) to identify risk factors of sleep abnormalities in non-sedated ICU patients 2) to describe the consequences of sleep disturbances on the outcome.

## Patients and Methods

This is an ancillary study of a single center randomized controlled trial. Inclusion criteria were:

1. no sedation for >24h,
2. sedation level < 3 on the Ramsay scale,
3. expected remaining ICU stay >48 hours,
4. morphine < 0.01 mg/kg/min,
5. norepinephrine < 0.3µg/Kg/min.

A 17h polysomnography was performed on the day of inclusion. Patients completed the Hospital and Anxiety depression scale at ICU and hospital discharge and at Day-90.

## Results

51 patients aged 64 [56-73] years were included. SAPS 2 was 37 [26-66]. Invasive or non-invasive ventilation was administered in 15 (29%) patients. ICU length of stay and mortality were 7 [3-13] days and 8% (n = 4). Total Sleep Time (TST) was 4.8 [3.2-6.8] hours including 13 [5-27] % stage 3+4 and 9 [1-18] % REM sleep. Nocturnal sleep accounted for 89 [76-98] % TST. Patients with a TST < 5 hours were more likely to have comorbidities as suggested a higher Charlson score (p = 0.008) and more frequent congestive heart failure (p = 0.04) and chronic respiratory disease (p = 0.04). TST < 5 hours was associated with more frequent nurse interventions (p = 0.03). Patients experiencing nocturnal REM sleep < 10% were more severe as shows their higher SAPS 2 (p = 0.01), had a higher length of ICU stay before inclusion (p = 0.04) and a lower comfort score at inclusion (p = 0.04). a TST < 5 hours was associated with more frequent depression at day-90 (p = 0.04). a nocturnal REM sleep < 10% was associated with a higher ICU length of stay (p = 0.02) and mortality (p = 0.04) and more frequent anxiety at ICU discharge (p = 0.03).

**Figure 1:**
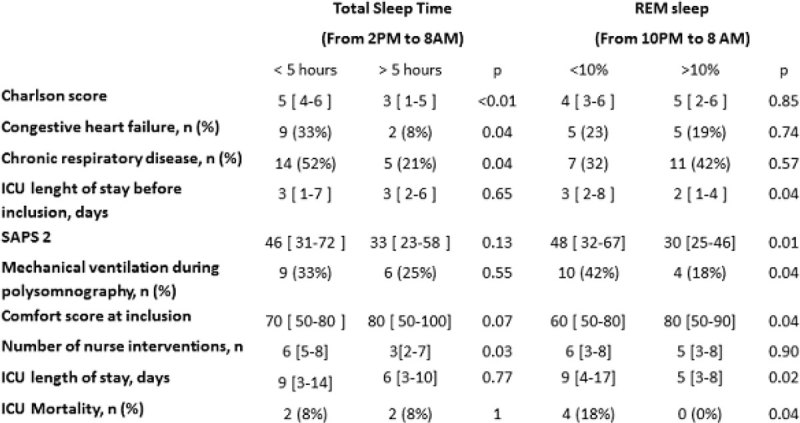
Figure 1

## Conclusions

Even in critically-ill patients who do not receive sedation, sleep quantity and architecture are severely altered. Comorbidities, severity of illness and human interventions negatively affect sleep quantity and architecture.

